# Hope or Anxiety for the Future? Associations of Climate Change Awareness and Hope With Eco‐Anxiety Among Nursing Students

**DOI:** 10.1111/inr.70195

**Published:** 2026-06-06

**Authors:** Merve Ataç Öksüz, Berna Akay, Dilek Aydin

**Affiliations:** ^1^ Faculty of Health Sciences Canakkale Onsekiz Mart University Canakkale Turkey; ^2^ Faculty of Health Sciences Alanya Alaaddin Keykubat University Antalya Turkey; ^3^ Faculty of Health Sciences Bandirma Onyedi Eylul University Balikesir Turkey

**Keywords:** awareness, climate change, eco‐anxiety, hope, nursing education, nursing students, planetary health

## Abstract

**Aim:**

To examine the effects of climate change awareness and hope on eco‐anxiety among nursing students.

**Background:**

Climate change is an escalating global health crisis affecting both physical and mental health, including eco‐anxiety. Understanding how awareness and hope shape nursing students’ emotional responses is essential for designing effective educational and support strategies.

**Methods:**

This cross‐sectional study was conducted with 312 nursing students in Turkey between March and June 2025. Descriptive statistics, Pearson's correlation, and hierarchical multiple regression analyses were performed.

**Results:**

Mean scores for the Hogg Eco‐Anxiety Scale, the Global Climate Change Awareness Scale, and the Climate Change Hope Scale were 18.13 ± 8.98, 70.68 ± 12.87, and 33.86 ± 7.23, respectively. Regression analysis revealed that environmental sensitivity (β = 0.17), membership in environmental organizations (β = 0.18), awareness of health effects (β = 0.13), perceived health impacts (β = 0.09), daily precautionary behaviors (β = 0.19), and higher climate change awareness (β = 0.37) were significant positive predictors of eco‐anxiety, whereas receiving climate change education (β = −0.17) and greater hope (β = −0.25) were significant negative predictors.

**Conclusion:**

While higher awareness was linked to increased eco‐anxiety, greater hope was related to reduced eco‐anxiety, suggesting that eco‐anxiety may play a dual role as both an emotional burden and a motivator for pro‐environmental engagement.

**Implications for nursing:**

Interactive, solution‐focused, and hope‐enhancing strategies should be integrated into nursing curricula to support students’ emotional adaptation to climate change.

**Implications for nursing policy:**

National nursing education policies should incorporate climate change competencies and psychosocial support frameworks to build a resilient and climate‐aware nursing workforce.

## 1 Background

Climate change is not merely an environmental issue but a multidimensional global crisis with far‐reaching implications for health systems, social equity, and mental well‐being (Romanello et al. 2023). The World Health Organization (WHO) identifies climate change as the greatest health threat to humanity, noting that the increasing frequency and severity of extreme events—such as heatwaves, floods, and wildfires—affect physical and mental health through both direct and indirect pathways (WHO 2023). The Intergovernmental Panel on Climate Change (IPCC) AR6 Synthesis Report highlights that current levels of warming already intensify climate‐sensitive health risks and deepen existing inequities, particularly among youth, older adults, and displaced populations (IPCC 2023).

Healthcare professionals—and nursing students as the future workforce—play a pivotal role in mitigating the health consequences of climate change (Nicholas et al. 2021). Therefore, strengthening nursing students’ climate‐related competencies is essential (International Council of Nurses [ICN] 2024). Planetary health competencies are increasingly recognized as core learning outcomes, encompassing climate‐resilient clinical care, effective risk communication, and advocacy (Jacobsen et al. 2024). Accordingly, integrating climate literacy into nursing curricula and enhancing students’ awareness and preparedness have become major educational priorities (Aronsson et al. 2024; Incesu and Yas 2024; Nicholas et al. 2021). Beyond technical and advocacy skills, it is equally vital to address the psychological dimensions through which climate change affects emotional functioning and mental health (Clayton et al. 2017; Pihkala 2020).

Climate change awareness—encompassing recognition of environmental threats and understanding their psychological and physiological effects—is a key component of professional competence in climate‐responsive nursing (ICN 2024; Nicholas et al. 2021). Such awareness can foster sensitivity, facilitate problem identification, and support effective responses (Deniz et al. 2021; Romanello et al. 2023). However, awareness is not purely cognitive; it may also evoke strong emotional reactions, including anxiety‐like responses, providing a psychological lens through which to understand eco‐anxiety (Clayton 2020; Kurth and Pihkala 2022).

Eco‐anxiety has emerged as a salient concept describing emotional–cognitive responses to environmental and climatic threats. It encompasses worry, sadness, guilt, and helplessness in response to perceived ecological degradation (Kurth and Pihkala 2022; Pihkala 2020). From an environmental stress perspective, heightened awareness can amplify perceived threat and emotional arousal, triggering eco‐anxiety that is shaped by individuals’ cognitive–motivational resources and coping mechanisms (Clayton et al. 2017; Ojala 2016; Pihkala 2020). Among young adults, eco‐anxiety is often linked with reduced hope for the future, diminished psychological flexibility, and impaired well‐being. Yet research suggests it is not inherently maladaptive; moderate concern can motivate constructive coping and pro‐environmental behavior, underscoring the importance of addressing eco‐anxiety without pathologizing it (Jarrett et al. 2024; Lau et al. 2024).

Within this dynamic, hope functions as a central psychological resource that can transform concern into constructive engagement. Snyder's hope theory conceptualizes hope as a goal‐directed cognitive–motivational construct consisting of agency and pathways components (Snyder 2002). In the climate context, constructive or critical hope—defined as realistic, solution‐oriented, and meaning‐focused—may help regulate negative affect and channel eco‐anxiety into adaptive coping and problem‐solving (Ojala 2012; 2023; Rizeq and Ojala 2025).

Among nursing students who may be both professionally and personally exposed to climate‐related risks, understanding how climate change awareness and hope are associated with eco‐anxiety is crucial for maintaining mental health and sustaining the quality of care. Although previous studies have examined links between climate change awareness and eco‐anxiety (Baykara Mat and Yilmaz 2024; İlaslan and Şahin Orak 2024), empirical evidence on the contribution of climate change hope to eco‐anxiety remains limited, particularly in nursing education contexts that increasingly emphasize planetary health competencies (Jacobsen et al. 2024; ICN 2024; Tümer et al. 2024). Addressing this gap is essential for informing psychosocial support and resilience‐focused interventions and for strengthening climate‐resilient competencies within nursing curricula. Accordingly, this study extends prior work by examining the associations among climate change awareness, climate change hope, and eco‐anxiety and by testing whether hope independently predicts eco‐anxiety beyond awareness and relevant demographic and climate‐related covariates.

### 1.1 Objectives

1. To assess the levels of eco‐anxiety, climate change awareness, and climate change hope among nursing students.
2. To examine the relationships among eco‐anxiety, climate change awareness, and climate change hope among nursing students.
3. To identify significant predictors of eco‐anxiety among nursing students.

## 2 Methods

### 2.1 Design

This cross‐sectional study was carried out at the nursing department of a state university in Turkey between March and June 2025. The Strengthening the Reporting of Observational Studies in Epidemiology (STROBE) guidelines were followed to enhance the rigor and transparency of the study reporting (von Elm et al. 2007).

### 2.2 Study Setting and Participants

The study was conducted at a public university in Turkey offering a four‐year undergraduate nursing program. The nursing curriculum includes courses such as Disaster Management and Nursing, Environmental Health, and Community Health Nursing, which cover issues related to planetary health, sustainable development, environmental risk management, and community‐based preparedness. These courses are offered as mandatory or elective components within the program and are scheduled across different academic years. This educational setting provides a relevant context for examining nursing students’ climate change awareness and emotional responses, including eco‐anxiety.

The study population consisted of 630 students enrolled in this undergraduate nursing program. Participants were recruited using a convenience sampling approach from a single institution. The minimum required sample size was estimated a priori using G*Power version 3.1.9.2 (Faul et al. 2009) for multiple linear regression (α = 0.05, medium effect size *f* ^2^ = 0.15, power = 0.80), with 13 predictors planned for inclusion in the final regression model, indicating a minimum sample size of 131 participants. The final analytic sample comprised 312 students who met the eligibility criteria.

Inclusion criteria were: (1) enrolment in the host university's undergraduate nursing program, (2) active course attendance during the data‐collection period, and (3) provision of informed voluntary consent. Exclusion criteria were: (1) enrolment in a non‐nursing or postgraduate program; (2) non‐attendance during the semester due to leave of absence, program freeze, or extension; (3) visiting/exchange student status (owing to contextual differences); (4) withdrawal of consent; and (5) data‐quality concerns (e.g., patterned responding, single‐option marking, or implausibly short completion times).

### 2.3 Measurements

Data were collected using four instruments: a Personal Information Form, the Hogg Eco‐Anxiety Scale (HEAS), the Global Climate Change Awareness Scale (GCCAS), and the Climate Change Hope Scale (CCHS).

#### 2.3.1 Personal Information Form

An investigator‐developed form was constructed with reference to the literature (Baykara Mat and Yilmaz 2024; İlaslan and Şahin Orak 2024; Tümer et al. 2024). The 11‐item instrument assessed demographic and climate‐related characteristics, including age, gender, income level, environmental sensitivity, membership in environmental organizations, and participation in climate change education. In this study, “received climate change education” was defined as having attended at least one formal course or structured educational session related to climate change. This definition included both university‐based courses and external educational activities such as workshops, seminars, or training sessions.

#### 2.3.2 Hogg Eco‐Anxiety Scale (HEAS)

The HEAS measures eco‐anxiety experienced over the past two weeks (Hogg et al. 2021). It consists of 13 items rated on a 4‐point Likert scale and includes four subscales: affective symptoms, behavioral symptoms, rumination, and anxiety about personal impact. Total scores range from 0 to 39, with higher scores indicating greater eco‐anxiety. The Turkish adaptation demonstrated excellent reliability (α = 0.91; Uzun et al. 2022), and internal consistency in this study was α = 0.94.

#### 2.3.3 Global Climate Change Awareness Scale (GCCAS)

The GCCAS assesses students’ awareness of global climate change (Deniz et al. 2021). It comprises 21 items rated on a 5‐point Likert scale and covers four domains: effects on the natural and social environment, global organizations and agreements, causes of climate change, and impact on energy consumption. Total scores range from 21 to 105, with higher scores reflecting greater awareness. Cronbach's α was 0.83 in the original study and 0.91 in this study.

#### 2.3.4 Climate Change Hope Scale (CCHS)

The CCHS evaluates climate‐related hope (Li and Monroe 2018; Gezer and İlhan 2020). It includes 11 items on a 5‐point scale with three subscales: personal‐sphere willpower and waypower, collective‐sphere willpower and waypower, and lack of willpower and waypower (reverse scored). Total scores range from 11 to 55; higher scores reflect greater hope. Cronbach's α was 0.87 in the Turkish adaptation and 0.93 in this study.

### 2.4 Procedure

Participants were informed about the study's purpose and procedures, assured of confidentiality, and reminded that they could withdraw at any time without penalty. All participants provided written informed consent. Data were collected using paper‐and‐pencil questionnaires administered by the researcher outside scheduled class hours, and completion took approximately 20 minutes. To preserve anonymity, no identifying information (e.g., names and student numbers) was recorded. Each participant was assigned a preprinted anonymous study ID (e.g., “NS‐247”) affixed to both the consent form and the questionnaire. Consent forms were stored separately from the questionnaires, and no linkage file or identifier connected them to the data set.

### 2.5 Ethical Considerations

The study was conducted in accordance with the ethical principles of the Declaration of Helsinki. Ethical approval was obtained from the Bandirma Onyedi Eylul University Health Sciences Non‐Interventional Research Ethics Committee (IRB approval number: March 10, 2025 /31). Institutional permission to conduct the study was also granted by the Dean of the Faculty of Health Sciences at Canakkale Onsekiz Mart University. Prior to data collection, all nursing students were informed about the aim of the study, the voluntary nature of participation, issues of confidentiality, and their right to withdraw at any time. Those who agreed provided written informed consent.

### 2.6 Data Analysis

Data were analyzed using IBM SPSS Statistics for Windows, Version 26.0 (IBM Corp., Armonk, NY, USA). The normality of the data distribution was tested using the Shapiro–Wilk test. Descriptive statistics are reported as frequencies (n), percentages (%), and means with standard deviations (M ± SD). Bivariate associations among eco‐anxiety, climate change awareness, and climate change hope were examined using Pearson's correlation coefficients. Predictors of eco‐anxiety were evaluated using a three‐step hierarchical multiple regression analysis. The block entry order was conceptually determined to reflect a progression from demographic and climate‐related covariates, to cognitive variables (awareness), and finally to psychological resources (hope). In Step 1, all demographic and climate‐related covariates were entered simultaneously as a single block, including age, gender, income level, environmental sensitivity, membership in an environmental organization, receipt of climate change education, awareness of climate change's health effects, perceived personal health impact of climate change, following climate change–related news, and daily precautionary behaviors. Prior to regression analysis, categorical predictors were dummy coded, and the reference categories are provided in the note to Table 1. In Step 2, the GCCAS total score was added to the model. In Step 3, the CCHS total score was entered. Incremental model fit was evaluated using ΔR^2^ and the associated ΔF statistics. Multicollinearity was assessed by examining variance inflation factors and tolerance values. Statistical significance was set at a two‐tailed *p* < 0.05 (Field 2024).

**TABLE 1:** Hierarchical regression coefficients predicting nursing students’ eco‐anxiety.

Predictor	*B*	*SE*	*β*	*t*	*p*	95% CI [LL, UL]
(Constant)	−0.82	4.55	—	−0.18	0.858	[−9.77, 8.14]
Age	0.04	0.18	0.01	0.25	0.802	[−0.30, 0.39]
Gender	−0.17	0.93	−0.01	−0.18	0.854	[−2.00, 1.66]
Low income	−2.36	1.51	−0.06	−1.56	0.119	[−5.32, 0.61]
High income	−0.90	0.92	−0.04	−0.97	0.331	[−2.72, 0.92]
Environmental sensitivity	3.40	0.77	0.17	4.44	< 0.001	[1.89, 4.91]
Membership in an environmental organization	3.59	0.91	0.18	3.95	< 0.001	[1.80, 5.38]
Received climate change education	−3.49	0.78	−0.17	−4.49	< 0.001	[−5.02, −1.96]
Awareness of climate change's health effects	2.63	0.81	0.13	3.24	0.001	[1.04, 4.23]
Personal impact of climate change on health	1.92	0.82	0.09	2.35	0.020	[0.31, 3.54]
Following climate change news	−0.75	0.84	−0.03	−0.89	0.377	[−2.40, 0.91]
Taking daily precautions against climate change	4.47	0.85	0.19	5.29	< 0.001	[2.81, 6.14]
GCCAS total	0.29	0.03	0.37	9.06	< 0.001	[0.23, 0.35]
CCHS total	−0.21	0.03	−0.25	−6.12	< 0.001	[−0.28, −0.14]

*Note*: Dependent variable: HEAS (Hogg Eco‐Anxiety Scale).

B: Unstandardized regression coefficient; β: Standardized regression coefficient; SE: Standard error; CI: Confidence interval; LL: Lower limit; UL: Upper limit; GCCAS: Global Climate Change Awareness Scale; CCHS: Climate Change Hope Scale.

Gender was coded as 0: female (reference); income was coded with 0: medium (reference); all other categorical predictors were coded as 0: no (reference).

## 3 Results

### 3.1 Nursing Students’ Characteristics

The sample comprised 312 nursing students meeting the inclusion criteria. The mean age was 21.58 years (SD = 2.07; range = 18–40). Most participants were female (83.9%) and rated their economic status as moderate (73.4%). In total, 44.2% described themselves as environmentally sensitive, 42.3% reported membership in an environmental organization, and 37.2% had received education on climate change. Nearly half (46.5%) reported awareness of the health effects of climate change, and 36.2% indicated that their physical or mental health had been directly affected. Moreover, 28.5% stated that they regularly follow climate change–related news, and 26.3% reported taking daily precautions to mitigate climate change.

### 3.2 Mean Scores on HEAS, GCCAS, and CCHS

The nursing students’ mean total scores were 18.13 ± 8.98 on the HEAS, 70.68 ± 12.87 on the GCCAS, and 33.86 ± 7.23 on the Climate Change Hope Scale (CCHS) (Table 2).

**TABLE 2:** Mean scores on HEAS, GCCAS, and CCHS.

Scales	Mean ± SD	Min–max	Score range
Hogg Eco‐Anxiety Scale	18.13 ± 8.98	2–39	0–39
Affective symptoms	5.70 ± 2.48	0–11	0–12
Behavioral symptoms	4.49 ± 2.54	0–9	0–9
Rumination	3.12 ± 1.85	0–8	0–9
Anxiety about one's personal impact	4.82 ± 2.24	0–9	0–9
Global Climate Change Awareness Scale	70.68 ± 12.87	40–98	21–105
Effects on the natural and social environment	37.02 ± 6.81	18–45	9–45
Global organizations and agreements	13.48 ± 6.49	6–28	6–30
Causes of global climate change	7.80 ± 3.43	3–15	3–15
Impact on energy consumption	12.38 ± 2.74	5–15	3–15
Climate Change Hope Scale	33.86 ± 7.23	11–54	11–55
Personal‐sphere willpower and waypower	9.18 ± 3.12	3–15	3–15
Collective‐sphere willpower and waypower	15.67 ± 5.60	5–25	5–25
Lack of willpower and waypower	9.01 ± 3.34	3–15	3–15

*Note*: HEAS: Hogg Eco‐Anxiety Scale; GCCAS: Global Climate Change Awareness Scale; CCHS: Climate Change Hope Scale.

### 3.3 Correlations Between HEAS, GCCAS, and CCHS

A strong, statistically significant positive correlation was observed between HEAS and GCCAS (*r* = 0.60, 95% CI [0.52, 0.67], *p* < 0.001), whereas HEAS was moderately and inversely correlated with CCHS (*r* = −0.47, 95% CI [−0.56, −0.37], *p* < 0.001).

### 3.4 Predictive Factors of Nursing Students’ Eco‐Anxiety

In Step 1 of the hierarchical multiple regression, demographic and environmental variables together explained 49.3% of the variance in eco‐anxiety (*R* ^2^ = 0.49, *p* < 0.001). Adding the GCCAS total score at Step 2 produced a significant increment (ΔR^2^ = 0.11, ΔF(1, 299) = 83.87, *p* < 0.001), increasing the explained variance to 60.4% (*R* ^2^ = 0.60). In Step 3, inclusion of the CCHS total score further improved the model (ΔR^2^ = 0.04, ΔF(1, 298) = 37.25, *p* < 0.001), yielding a final model that explained 64.8% of the variance in eco‐anxiety (*R* ^2^ = 0.65; Table 3).

**TABLE 3:** Hierarchical regression models predicting eco‐anxiety.

Model	*R*	*R* ^2^	Adj. *R* ^2^	SE	Δ*R* ^2^	Δ*F*	*df*_1_/*df*_2_	*p*
1	0.70	0.49	0.47	7.24	—	—	—	—
2	0.78	0.60	0.59	6.41	0.11	83.87	1/299	< 0.001
3	0.81	0.65	0.63	6.06	0.04	37.25	1/298	< 0.001

*Note*: Dependent variable: Hogg Eco‐Anxiety Scale (HEAS).

R: Multiple correlation coefficient; *R* ^2^: Coefficient of determination; Adj. *R* ^2^: Adjusted *R* ^2^; SE: Standard error of the estimate; Δ*R* ^2^: *R* ^2^ change; ΔF: F change.

*df* values correspond to the ΔF test at each step (*N* = 312).

Model 1 includes demographic and climate‐related variables; Model 2 adds the Global Climate Change Awareness Scale (GCCAS); Model 3 adds the Climate Change Hope Scale (CCHS).

The final model (Model 3) was statistically significant (*p* < 0.001). Age, gender, and income level were not significant predictors (*p* > 0.05). In contrast, environmental sensitivity (β = 0.17, *p* < 0.001), membership in an environmental organization (β = 0.18, *p* < 0.001), awareness of the health effects of climate change (β = 0.13, *p* = 0.001), personal impact of climate change on health (β = 0.09, *p* = 0.02), taking daily precautions against climate change (β = 0.19, *p* < 0.001), and GCCAS total score (β = 0.37, *p* < 0.001) were significant positive predictors. Conversely, receiving education on climate change (β = –0.17, *p* < 0.001) and CCHS total score (β = –0.25, *p* < 0.001) were significant negative predictors (Table 1).

## 4 Discussion

This study examined the relationships among climate change awareness, climate change hope, and eco‐anxiety in nursing students, adjusting for demographic and climate‐related covariates. Overall, students’ eco‐anxiety levels fell within the low‐to‐moderate range, consistent with comparable samples in the literature (Baykara Mat and Yilmaz 2024; Er et al. 2024; Tümer et al. 2024). Subscale analyses indicated relatively higher affective symptoms and anxiety about personal impact, alongside lower rumination and behavioral symptoms. This pattern suggests that the threat is perceived at a personal level, yet the anxiety has not evolved into persistent cognitive preoccupation or pronounced behavioral symptoms; accordingly, it reflects a functional/manageable affective state. This profile aligns with approaches that conceptualize eco‐anxiety as an adaptive response to environmental threat rather than a pathological syndrome; in many contexts, low‐to‐moderate anxiety may heighten attention and action tendencies without eroding subjective well‐being (Boluda‐Verdú et al. 2022). However, when anxiety becomes chronic, overwhelming, or disengaging, it may shift from an adaptive to a maladaptive form—reducing motivation and impairing functioning (Clayton 2020; Pihkala 2020). Distinguishing between these forms is essential for nursing educators to recognize when eco‐anxiety can be harnessed for engagement and when it requires psychological support. Within nursing education, the systematic integration of climate–health scenario‐based simulations, brief reflective sessions, and solution‐ and hope‐focused modules could channel this low‐to‐moderate eco‐anxiety into constructive engagement, thereby strengthening students’ climate literacy and action self‐efficacy (ICN 2024; Levett‐Jones et al. 2025; Tiitta et al. 2024).

In the current study, climate change awareness was generally moderate to high, consistent with prior work (Incesu and Yas 2024; Schenk et al. 2021; Tümer et al. 2024). Subscale findings showing higher awareness of “effects on the natural and social environment” suggest that students construe the threat primarily through macro‐level outcomes—such as ecosystem imbalance, disaster risk, biodiversity loss, and social inequalities—rather than exclusively through individual consequences (Clayton et al. 2017; IPCC 2023). Moreover, a positive correlation between global climate change awareness and eco‐anxiety was observed, and this association remained significant in hierarchical models after controlling for demographic characteristics and climate‐related behaviors. This result aligns with previous studies reporting that greater awareness of environmental threats is associated with higher eco‐anxiety among nursing students (Baykara Mat and Yilmaz 2024; İlaslan and Şahin Orak 2024; Tümer et al. 2024). Theoretically, this relationship can be interpreted through environmental stress theory, which posits that increased knowledge of environmental risks heightens perceived vulnerability, thereby amplifying emotional and cognitive stress responses (Clayton et al. 2017; Helm et al. 2018). Contemporary models further suggest that the adaptiveness of eco‐anxiety depends on coping resources and distress intensity (Clayton 2020). Given nursing education's dual emphasis on health promotion and environmental responsibility, rising awareness may evoke a sense of professional/moral responsibility and urgency, which for some students may co‐occur with heightened eco‐anxiety (Aronsson et al. 2024; ICN 2024). Accordingly, educational approaches that pair heightened awareness with action pathways and coping resources may help translate concern into constructive engagement (Finnegan and d'Abreu 2024; Lee et al. 2024; Tiitta et al. 2024). Importantly, climate change hope may buffer awareness‐linked eco‐anxiety and influence whether it is channeled into action‐oriented coping and professional engagement or instead becomes disengaging distress (Tümer et al. 2024).

In this study, nursing students reported moderate levels of climate change hope, consistent with previous findings (Baykara Mat et al. 2024; Tümer et al. 2024). Hope was negatively correlated with eco‐anxiety, and in hierarchical models, it remained an independent negative predictor of anxiety. This pattern indicates that as expectations for solutions and action increase, the intensity of anxiety decreases. The literature suggests that hope is associated with reduced negative affect and facilitates meaning‐focused coping, thereby supporting subjective well‐being and pro‐environmental behavior (Li and Monroe 2019; Ojala 2012; Rizeq and Ojala 2025). Nevertheless, some studies among nursing students have reported a positive correlation between climate concern and hope (Baykara Mat et al. 2024; Tümer et al. 2024). This variability may reflect differences in conceptualization and measurement, for example, whether studies assess general or climate‐specific hope, or broader worry rather than eco‐anxiety, as well as contextual influences. Accordingly, rather than emphasizing information transmission alone, nursing curricula should prioritize solution‐oriented, hope‐infused learning experiences to keep anxiety at manageable levels and enhance professional engagement (Finnegan and d'Abreu 2024; Lee et al. 2024; Tiitta et al. 2024). Such pedagogical alignment is consistent with the International Council of Nurses’ (ICN) planetary health competencies and the United Nations Educational, Scientific and Cultural Organization's (UNESCO) framework for sustainability education, both of which advocate integrating emotional resilience, reflective capacity, and systems thinking into nursing curricula (ICN 2024; UNESCO 2020). In particular, strengthening the agency and pathways components of hope may lessen feelings of helplessness or overwhelm by framing climate action as a shared and attainable goal (Ballew et al. 2024; Ojala 2012).

In the present study, environmental sensitivity, membership in environmental organizations, awareness of climate‐related health effects, perceived personal health impact, and daily precautionary behaviors emerged as significant predictors of eco‐anxiety. The literature indicates that environmental concern can be positively associated with both individual pro‐environmental behaviors and collective action (Ballew et al. 2024; Baroni et al. 2025; Ogunbode et al. 2022). This pattern suggests that individuals with a heightened sense of threat tend to show stronger commitment to environmental protection; however, perceived personal impact, increased exposure to information, and a heightened sense of individual responsibility may also elevate anxiety levels (Helm et al. 2018; Reese et al. 2023). At the same time, constructive forms of habitual ecological worry can support environmental action and foster active coping rather than passive helplessness (Verplanken and Roy 2013). Accordingly, eco‐anxiety should be framed not only as an emotional burden but also as a potential motivational driver that nurtures environmental commitment and proactive orientations. To keep this emotional response at manageable levels, nursing curricula should integrate applied, interactive learning experiences that build students’ hope, self‐efficacy, and collective action skills (Finnegan and d'Abreu 2024; Ojala 2012; Tiitta et al. 2024).

Another salient finding was that having received climate change education was associated with lower eco‐anxiety. By contrast, Çolak et al. (2025) reported that, among nursing students, eco‐anxiety scores increased following a climate change and health course despite gains in knowledge and attitudes. This finding suggests that the emotional tone of educational content can substantially influence its psychological outcomes. The discrepancy implies that curricula focusing primarily on information transmission may at times heighten perceived threat, whereas programs that strengthen coping skills, critical thinking, and self‐efficacy are more likely to reduce maladaptive anxiety. Consistent with this interpretation, Olsen et al. (2024) report that action‐oriented, empathy‐supported, structured educational interventions strengthen the functional dimension of eco‐anxiety while mitigating its adverse effects. For nursing faculty, these components can be operationalized through reflective writing exercises, simulation‐based discussions, and peer dialog sessions (Finnegan and d'Abreu 2024; Ojala 2023; Olsen et al. 2024).

### 4.1 Limitations

To our knowledge, studies examining both climate change awareness and hope as predictors of eco‐anxiety among nursing students remain limited. By addressing this gap, the present study contributes to the emerging evidence base; however, several limitations should be considered when interpreting the findings. Although the study identified multiple significant predictors of eco‐anxiety, its cross‐sectional design precludes any inference about the causal direction between awareness, hope, and anxiety. Furthermore, the associations among these variables were based on self‐reported perceptions, which may have been influenced by participants’ subjective interpretations or current emotional states. Despite all eligible students being invited to participate, recruitment relied on self‐selection, which may have introduced selection bias and should be taken into account when interpreting the generalizability of the findings. In addition, conducting the study within a single cultural and educational context may have influenced the strength and direction of the observed associations, thereby limiting the transferability of the results to other populations. The predominantly female composition of the sample (83.9%) further constrains generalizability to male students or more gender‐balanced cohorts, given potential gender differences in emotional responses to climate change. Moreover, the use of self‐report measures is inherently susceptible to recall bias and social desirability bias. Finally, restricting the sample to nursing students limits the applicability of the findings to other health‐related disciplines and broader student populations.

Future research should therefore include cross‐cultural validation and comparisons (e.g., across countries or nursing curricula) to enhance external validity and examine whether the observed relationships hold across diverse educational and sociocultural settings. Moreover, multicenter, longitudinal, or interventional designs should be employed to test temporal and causal mechanisms, including potential mediation or moderation effects—for example, whether hope mediates the relationship between climate change awareness and eco‐anxiety. Finally, qualitative or mixed‐method studies could provide deeper insights into students’ lived experiences, contextual meanings, and coping processes related to climate‐induced emotional responses. Collectively, such studies may inform evidence‐based educational strategies that enhance psychosocial well‐being and support the sustainability of climate‐responsive nursing education.

## 5 Conclusions

This study showed that climate change awareness was positively associated with eco‐anxiety, whereas climate change hope functioned as an independent protective factor, predicting lower levels of eco‐anxiety even after controlling for awareness and other covariates. In addition, environmental sensitivity, membership in environmental organizations, awareness of climate change's health effects, perceived personal health impact, and daily precautionary behaviors were significant positive predictors of eco‐anxiety, while having received climate change education was linked to lower levels. Overall, these findings highlight the dual nature of eco‐anxiety, which may emerge as an emotional burden following increased awareness, yet may be channeled into adaptive coping and sustained pro‐environmental action when accompanied by hope.

### 5.1 Implications for Nursing

The findings underscore the importance of integrating climate change and planetary health competencies into nursing curricula through interactive, solution‐oriented, and hope‐enhancing approaches. Such strategies can help channel moderate eco‐anxiety into constructive engagement while simultaneously supporting students’ psychosocial well‐being. Curriculum initiatives should move beyond information transmission to include coping strategies, critical appraisal skills, and opportunities for collective action, thereby enhancing both emotional resilience and professional competence in climate‐responsive care. Furthermore, embedding systematic evaluation and assessment mechanisms within curricula can ensure sustainable, evidence‐informed educational practices. An integrated model that addresses both the cognitive and affective dimensions of climate change may foster a resilient, knowledgeable, and proactive health workforce capable of advancing climate‐responsive health systems.

## Author Contributions

Study design: MAÖ, BA, and DA. Data collection: MAÖ. Data analysis: DA and MAÖ. Study supervision: DA and MAÖ. Manuscript writing: MAÖ, BA, and DA. Critical revisions for important intellectual content: MAÖ, BA, and DA.

## Conflicts of Interest

The authors declare no conflicts of interest.

## Funding

The study was funded by the Bandirma Onyedi Eylul University.

## Data Availability

Data are available upon reasonable request, by sending an e‐mail to the corresponding author.
